# The leprosy reaction is associated with salivary anti-*Porphyromonas gingivalis* IgA antibodies

**DOI:** 10.1186/s13568-023-01576-1

**Published:** 2023-07-07

**Authors:** Michelle Miranda Lopes Falcão, Johelle Santana Passos-Soares, Paulo Roberto Lima Machado, Isaac Suzart Gomes-Filho, Lucas Pedreira de Carvalho, Elisangela Jesus de Campos, Mariana Costa Calheira, Patrícia Mares de Miranda, Rebeca Pereira Bulhosa Santos, José Tadeu Raynal Rocha Filho, Antonio Pedro Froes de Farias, Taiana Peixoto, Roberto Meyer Nascimento, Gregory John Seymour, Soraya Castro Trindade

**Affiliations:** 1 Department of Health, State University of Feira de Santana, Feira de Santana, Brazil; 2 Postgraduate Program in Immunology, Federal University of Bahia, Salvador, Brazil; 3 Preventive Dentistry Department, Federal University of Bahia, Salvador, Brazil; 4 Immunology Service, University Hospital Prof. Edgar Santos Federal University of Bahia, Salvador, Brazil; 5 Immunology and Molecular Biology Laboratory, Federal University of Bahia, Salvador, Brazil; 6 Oral Biochemistry Laboratory, Health Sciences Institute, Federal University of Bahia, Salvador, Brazil; 7 School of Dentistry, Federal University of Bahia, Salvador, Brazil; 8 School of Dentistry, The University of Queensland, Brisbane, Australia

**Keywords:** Saliva, Periodontitis, Humoral response, Leprosy

## Abstract

The aim of the study was to evaluate the association between salivary anti-*Porphyromonas gingivalis* IgA antibodies and the leprosy reaction. The levels of salivary anti - *P. gingivalis* IgA antibodies, together with salivary flow and pH were measured in individuals diagnosed with leprosy and associated with the development of the leprosy reaction. Saliva was collected from 202 individuals diagnosed with leprosy at a reference leprosy treatment center, 106 cases with the leprosy reaction and 96 controls without the leprosy reaction. Anti - *P. gingivalis* IgA was evaluated by indirect immunoenzyme assay. Non-conditional logistic regression analysis was employed to estimate the association between antibody levels and the leprosy reaction. There was a positive statistically significant association between the levels of anti - *P. gingivalis* IgA and the presence of the leprosy reaction, controlling for confounders: age, sex, level of education and alcoholic beverage consumption: OR_ajusted_: 2.55; IC 95%: 1.34–4.87. Individuals with leprosy who had high levels of salivary anti - *P. gingivalis* IgA had approximately twice as many chances of developing the leprosy reaction. The findings suggest a possible relationship between salivary anti - *P. gingivalis* IgA antibodies and the leprosy reaction.

## Key Points

- Oral manifestations may predispose to the leprosy reaction.
- Salivary anti - *P. gingivalis* IgA antibodies are associated with leprosy reaction.
- Marker for leprosy reaction in individuals with periodontitis.

## Introduction

The leprosy reaction is an acute inflammatory condition that occurs in about 40% of leprosy patients. It usually occurs before or at the beginning of treatment but can occur at any stage of the disease or multidrug therapy, including some years after the conclusion of the treatment (Graham et al. 2010). The leprosy reaction may be classified as a type 1 or Reversal Reaction or type 2, also known as Erythema Nodosum Leprosum. The type 1 reaction is characterized by delayed hypersensitivity with involvement of cellular immunity and a clinical presentation of a skin erythematous plaque accompanied by pain, edema, and neural sensitivity, usually without systemic involvement (Lockwood and Saunderson 2012). The type 2 reaction involves humoral immunity and immune complex formation, resulting in multiple nodules in the skin, including necrosis and the presence of systemic manifestations, such as fever, muscle and joint pain, neuritis and peripheral adenopathy (Polycarpou et al. 2017). There is a type of reaction known as de Lucio’s phenomenon or type 3 reaction that is characterized by the development of severe, necrotizing, cutaneous lesions with a high mortality rate. This phenomenon is rare and occurs in patients with diffuse lepromatous leprosy (Frade et al. 2022).

The etiology of leprosy reactions has not yet been well elucidated, but it is believed that concomitant infections, including oral infections, pregnancy, puerperium, use of iodinated drugs, or physical and emotional stress might be risk factors for developing leprosy reactions, with putative immune instability being responsible for the appearance and maintenance of leprosy (Browne 1963; Lockwood and Sinha 1999; Motta et al. 2012).

Leprosy is caused by *Mycobacterium leprae* and is more prevalent in developing countries. It is considered a public health problem in Brazil, with a prevalence of 104.6/ million population (WHO 2021). A higher occurrence of periodontitis has been observed in individuals with leprosy (Ranganathan et al. 2014; Raja et al. 2016) and periodontal treatment has been shown to reduce the susceptibility to leprosy reaction in individuals with leprosy under multidrug therapy (Motta et al. 2011). In this context it can be speculated that the periodontal infection may be one of the factors that initiate or exacerbate the leprosy reaction by increasing the systemic inflammatory load (Motta et al. 2011; Cortela et al. 2015).

Periodontitis is the 4th public health burden worldwide (GBD 2015). and has been associated with several systemic conditions (Willis and Gabaldón 2020), which are interlinked through sharing underlying infection and inflammatory pathways (Jin et al. 2016). The etiology is complex, multifactorial, and polymicrobial, dependent on the genetic susceptibility of the host; the response of the immune system to the microbial challenge (Hajishengallis 2014); and the presence of an oral dysbiotic biofilm with the participation of keystone pathogens, such as *P. gingivalis* (O’Brien-Simpson et al. 2000; Olczak et al. 2008, 2010; Santos-Lima et al. 2020).

The presence of antibodies specific to *P. gingivalis* has been claimed to be a marker of periodontitis (Shah et al. 2010; Gao et al. 2020). These molecules are present in saliva and may in part be due to the severity of periodontal inflammation and the concomitant increased flow of gingival crevicular fluid containing both serum and locally produced antibodies. However, the predominant immunoglobulin in saliva is secretory-IgA which is due to mucosal presentation of the organism, and which may or may not be indirectly related to the severity of periodontal inflammation (Shah et al. 2010; Isola et al. 2020). In addition, regular salivary flow and pH stability are associated with the buffering capacity of the environment, consequently the stability of the oral microbiome (Rosier et al. 2018), whose dysbiosis has already been related to periodontitis (Hajishengallis 2015).

These oral manifestations may represent disturbances in the immune response which in turn may predispose to the leprosy reaction. This study therefore evaluated the association between anti - *P. gingivalis* IgA salivary and the leprosy reaction. Further, saliva can be obtained with a simple and easy method for diagnosis, not requiring invasive techniques. To maintain reliability and comparison between the investigated groups, salivary pH and flow were evaluated. The hypothesis of the present study is that high levels of salivary anti - *P. gingivalis* IgA antibodies are associated with the leprosy reaction.

## Materials and methods

### Sample selection

A case-control study was carried out on individuals diagnosed with leprosy, followed up in the dermatology clinic of the Professor Edgard Santos University Hospital from December 2015 to February 2020. Individuals with leprosy reactions were considered cases and those without leprosy reactions were the controls. The individuals selected to compose the control group were recruited from the same health service and at the same time as those in the case group. The sample size was calculated from the standard deviation and mean IgA levels specific for *P. gingivalis* at 0.28 and 0.52, respectively. These values were obtained in the pilot study previously conducted at the 95% significance level and with 10% acceptable error, which resulted in 111 participants in each group.

This research was approved by the Research Ethics Committee of the Feira de Santana State University (CAEE: 47218315.3.0000.0053) and all individuals who agreed to participate signed the consent form. Only individuals over the age of 18, with a diagnosis of leprosy, who had at least four teeth, and who had not received treatment for periodontitis in the past 6 months were recruited for the study. Individuals with leprosy had the diagnosis performed by dermatologists (Brasil 2016, 2017). They were those with thickened peripheral nerves and/or skin lesions or areas of skin with thermal and/or painful and/or tactile sensitivity changes (WHO 2017). Pregnant women, individuals with neoplasia or HIV-AIDS, absence of a histopathological diagnosis of leprosy missing about the patient’s health condition in the medical record did not participate in the study, as did individuals who were unable to answer the questionnaires and with irregular adherence to treatment.

A questionnaire containing information on economic and socio-demographic condition, health condition and lifestyle was applied at the beginning of the data collection to obtain information on the general characteristics of the comparison groups.

### Diagnosis of leprosy reaction

Diagnosis of a leprosy reaction was performed by dermatologists (Brasil 2016, 2017). Individuals with erythematous skin lesions, with the presence of edema and pain associated with paresthesia at sites infected with *M. leprae* were diagnosed with a type 1 reaction. And individuals who developed painful subcutaneous nodules and erythematous associated with systemic manifestations, such as fever (WHO 2017) were diagnosed with type 2 reaction.

### Collection and evaluation of pH and saliva flow

Following fasting for 2 h saliva collection was performed after mechanical stimulation with parafilm chewing (Krasse 1988; Navazesh 1993; Navazesh and Kumar 2008).

Salivary flow was determined by measuring the total amount produced over 5 min. It was considered normal if the flow rate was between 1.0 and 3.0 mL/min; low if the flow rate was between 0.7 and 0.9 mL/min; and hyposalivation was identified if the flow rate was between 0.5 and 0.6 mL/min.

One mL of saliva was mixed with 3 mL of HCl at 0.005% and after 10 min the pH was measured a QUIMIS Q400-A pHmeter (QUIMIS, Diadema, SP). The reference value adopted for normal buffer capacity was a final pH between 5.0 and 7.0 and for low buffer capacity, a final pH of 4.0 (Thylstrup and Fejerskov 1995; Dawes 2008). The remaining saliva was stored in an Eppendorf tube and centrifuged, the supernatant removed and stored at − 70 °C with 1 mL protease inhibitor (SIGMA-ALDRICH, Saint Louis, USA) until further analysis.

### Immunoenzymatic assay

The immunoenzymatic assay (ELISA) was used to evaluate the salivary levels of IgA against antigens present in the sonic extract of *P gingivalis* from the ATCC33277 strain. The assays were performed using high adsorption polystyrene plates with 96 flat bottom wells (Greiner Bio-One, Frickenhausen, Germany). Following the conditions obtained after standardization of the assay (Calheira et al. 2021), the wells were sensitized with *P. gingivalis* antigens − 0.65 µg/mL (Trindade et al. 2008) diluted in carbonate buffer and incubated in a humidity chamber at 8 °C for 15 h. They were then washed twice with phosphate buffered saline (Phosphate Buffered Saline-PBS) 0.15 M, pH 7.4, containing 0.05% Tween 20 (PBS-T) and blocked with skim milk powder (MOLICO^®^, Nestle, Brazil) diluted at 5% in 0.15 M PBS, pH 7.4 and incubated at 37 °C for 2 h.

After further washing with PBS-T and drying, the plates with the samples of saliva diluted at 1:50 in 2% skim milk (MOLICO^®^, Nestle, Brazil) (PBS 0.15 M, pH 7.4) were incubated for another hour. After five washes with PBS-T and drying, goat anti-human IgA conjugated to a peroxidase (INVITROGEN, Frederick, USA), diluted at 1:5000, was inserted into each well and the plate incubated for a further one hour. Following, five more PBS-T washes peroxidase was developed using tetramethylbenzidine chromogen (TMB) (ABCAM, California, USA) for approximately 5 min. The reaction was stopped with sulfuric acid, (H_2_SO_4_)4 N. The optical densities (OD) were measured by spectrophotometry using an ELISA reader (Multiskan GO, Term Fisher Scientific OY, Vantaa, Finland). The data from the spectrophotometric readings were transferred to an EXCEL for Windows® spreadsheet, processed and corrected (Zwirner 1996).

### Statistical analysis

The descriptive analysis of the data was carried out using absolute frequencies, percentages, and measures of central tendency. To verify the statistical differences between the case and control groups, Pearson’s Chi-square and Fisher’s Exact tests for the categorical variables and Mann Whitney’s for the continuous variables were used in the bivariate analysis. IgA levels were grouped using the cutoff point of 0.134, according to Calheira et al. (2021). The data regarding pH and salivary flow were categorized, being considered reduced salivary flow ≤ 1.0 mL/min and normal > 1.0 mL/min and acid pH < 6.8 and normal ≥ 6.8.

Then, unconditional logistic regression analysis was applied to examine the association between immunoglobulin levels studied and the leprosy reaction, using the Backward strategy, considering as confounders the covariates age, sex, education level and alcohol consumption. All analyses were performed at IBM SPSS Statistics 23, adopting the significance level of 5%.

## Results

A total of 475 people were invited to participate in the study, however, only 202 satisfied the eligibility criteria, of which 96 made up the control group and 106 the case group: 49 with a type 1 reaction and 57 with a type 2 reaction. The comparison groups were similar regarding socioeconomic-demographic covariables, health condition and lifestyle, except for education level (p < 0.001), household density (p = 0.02), alcoholic beverage consumption (p = 0.01) and daily brushing frequency (p = 0.04) (Table 1).

**Table 1:** Distribution of socioeconomic-demographic characteristics, health conditions and lifestyle according to the presence (cases) and absence (controls) of leprosy reaction

Variable	Without reaction		With reaction		*P* value
	n	%	n	%	
Age (n = 202)					
≤ 50 years	52	54.2	59	55.7	0.83
> 50 years	44	45.8	47	44.3	
Sex (n = 202)					
Male	55	57.3	71	67.0	0.16
Female	41	42.7	35	33.0	
Skin tone (n = 198)					
White	12	12.6	11	10.7	0.67
No white	83	87.4	92	89.3	
Level of education (n = 200)					
≤ 12 years of study	40	41.7	74	69.8	< 0.001
> 12 years of study	56	58.3	32	30.2	
Marital status (n = 201)					
Living with partner	49	51.0	57	54.3	0.65
Living without partner	47	49.0	48	45.7	
Household density (n = 202)					
≤ 3 people/household	75	78.1	67	63.2	0.02
> 3 people/household	21	21.9	39	36.8	
Hypertension (n = 201)					
No	70	72.9	73	69.5	0.60
Yes	26	27.1	32	30.5	
Diabetes (n = 201)					
No	89	92.7	91	86.7	0.16
Yes	07	7.3	14	13.3	
Smoking habit (n = 198)					
No	84	88.4	91	88.3	0.10
Yes	11	11.6	12	11.7	
Alcoholic beverage consumption (n = 194)					
No	65	69.9	86	85.1	0.01
Yes	28	30.1	15	14.9	
Visit to dentist (n = 201)					
No	05	5.2	08	7.6	0.57
Yes	91	94.8	97	92.4	
Guidance on oral hygiene (n = 199)					
No	34	35.4	40	38.8	0.62
Yes	62	64.6	63	61.2	
Daily brushing (n = 200)					
≥ 2 times/day	92	95.8	91	87.5	0.04
< 2 times/day	04	4.2	13	12.5	
Use of dental floss (n = 200)					
Yes	52	54.7	51	48,6	0.38
No	43	45.3	54	51.4	

With respect to oral conditions , there was no statistically significant differences between the groups with and without the leprosy reaction in the number of teeth in the mouth (p = 0.168), periodontal pocket probing depth (p = 0.152), bleeding on probing (p = 0.247) and number of teeth with CAL ≥ 5 mm (p = 0.114). Participants with the leprosy reaction showed a greater number of teeth with CAL = 3 and 4 mm (p = 0.004) and a smaller number of teeth with CAL = 1 and 2 mm (p = 0.005).

A positive association between high levels of IgA anti - *P. gingivalis* and the leprosy reaction was observed in the unadjusted model (OR_unadjusted_: 2.24; 95% CI 1.25–4.00) and in the model adjusted for the covariables age, sex, education level and alcoholic beverage consumption (OR_adjusted_: 2.55; 95% CI 1.34–4.87), as can be observed in Table 2.

**Table 2:** Association measurements between immunoglobulin A anti-*Porphyromonas gingivalis* and leprosy reaction

Model	Odds Ratio	95% Confidence interval	*P* ^b^
Leprosy reaction x IgA anti - *P. gingivalis*			
Unadjusted	2.24	1.25–4.00	0.006
Adjusted ^**a**^	2.55	1.34–4.87	0.005

^a^Adjustment by age, sex, education level and alcoholic beverage consumption

^b^Statistical significance: p ≤ 0.05

**Fig. 1:**
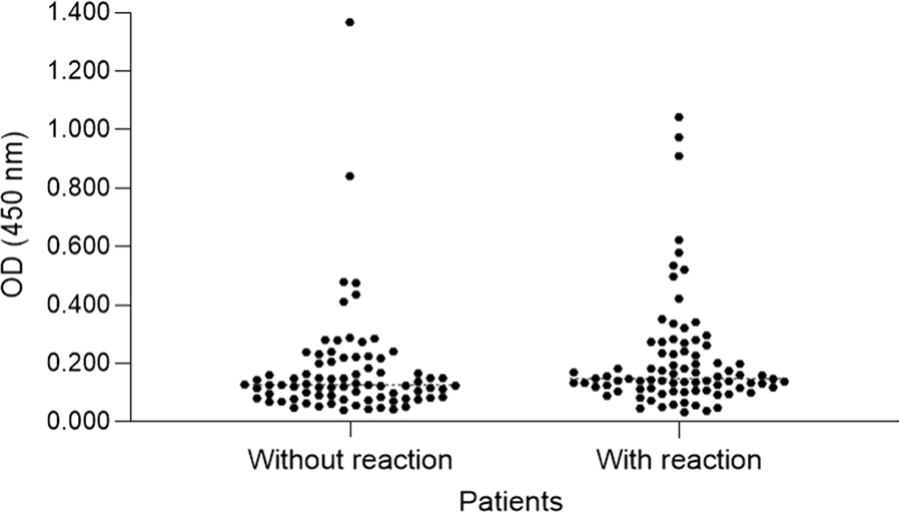
Distribution of the optical density (OD) value of IgA anti-*P. gingivalis* I between patients with and without leprosy reaction

**Fig. 2:**
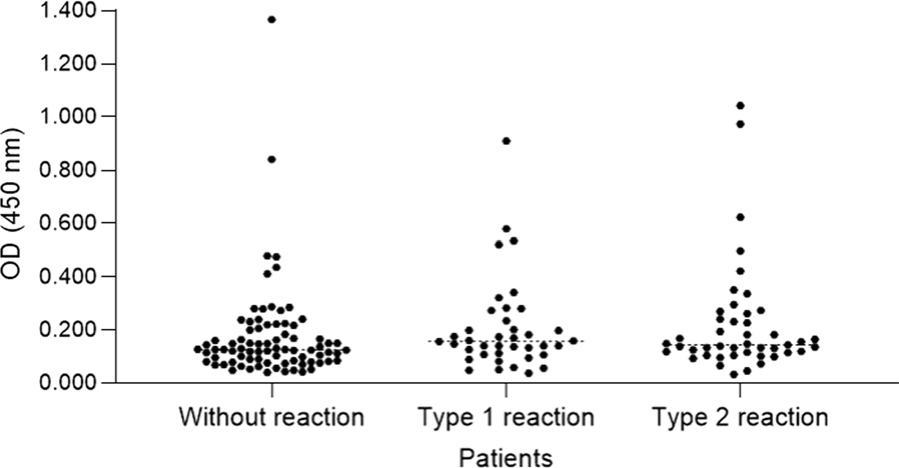
Distribution of the optical density (OD) value of IgA anti-*P. gingivalis* among the different types of leprosy reaction

**Fig. 3:**
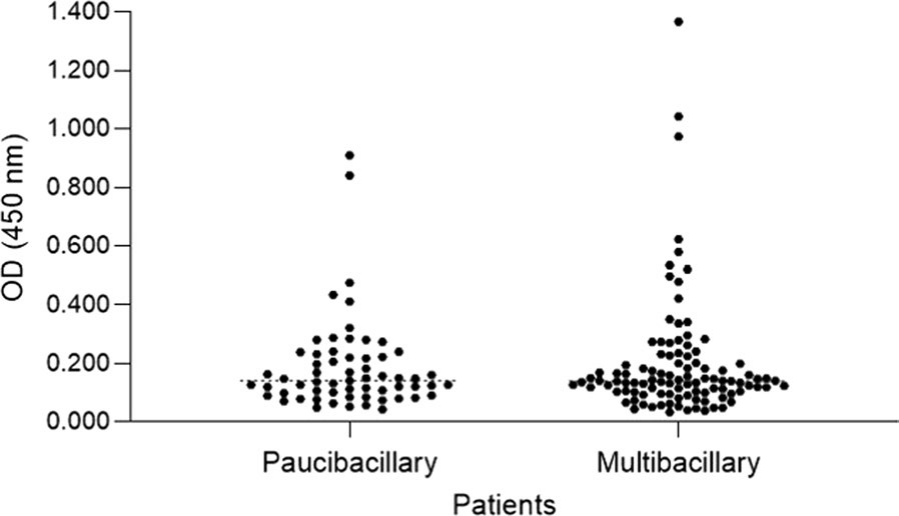
Distribution of the optical density (OD) value of IgA anti - *P. gingivalis* among the different types of leprosy

There was no significant statistical difference between the case group and the control group in saliva production (p = 0.62) and salivary pH (p = 0.18). The median saliva production in the case group was 0.88 mL/m (IQR: 0.58–1.37) and in the control group 0.80 mL/m (IQR: 0.52–1.26). The median pH in the case group was 7.03 (IQR: 6.75–7.12) and in the control group 6.92 (IQR: 6.59–7.11). Figures 1, 2 and 3 show the distribution of the optical density (OD) value of IgA anti - *P. gingivalis* among the different groups.

The absence of statistical significance was maintained between the control group and the case group divided according to the type of reaction. The median salivary flow of the group with a type 1 reaction was 0.98 mL/m (IQR: 0.77–1.49; p = 0.06) and the group with a type 2 reaction was 0.80 mL/m (IQR: 0.43–1.20; p = 0.40). The median pH of the group with a type 1 reaction was 6.99 (IQR: 6.80–7.11; p = 0.25) and of the group with a type 2 reaction was 7.04 (IQR: 6.72–7.15; p = 0.30). However, the group with a type 1 reaction presented higher salivary flow than the group with a type 2 reaction (p = 0.03).

Considering the cutoff point ≥ 1mL/minute for normal salivary flow, 45.8% (n = 44) of the individuals in the control group presented reduced salivary flow, while this percentage in the group with the type 1 reaction was 30.6% (n = 15) and in the group with the type 2 reaction it was 40.4% (n = 23). These differences were not statistically significant (data not shown). A salivary pH above 7 was shown in more than half of the samples from each group; 58.3% (n = 56) of those without the reaction, 71.7% (n = 76) of those with the reaction, 69.4% (n = 34) of those with a type 1 reaction and 73.7% (n = 42) of those with a type 2 reaction.

## Discussion

The results of the present study have shown that individuals with leprosy who had high levels of salivary anti - *P. gingivalis* IgA antibodies had approximately twice as many chances of having a leprosy reaction. If the presence of anti-*P. gingivalis* IgA antibodies in saliva is considered as an indirect indicator of periodontitis, these findings may be consistent with the scarce literature, which found that periodontal disease, the presence of periodontal pockets and bone loss, and oral infections are associated with the leprosy reaction (Motta et al. 2010, 2011; Cortela et al. 2015).

Leprosy reactions have among their etiological components an imbalance in the immunological mechanism involved in the development and maintenance of inflammatory disorders. As periodontitis is a manifestation of the systemic immune system, high levels of salivary (secretory) anti - *P. gingivalis* IgA antibodies may reflect an imbalance between the systemic and mucosal immune systems which in turn may predispose to the leprosy reaction.

The detection of anti-*P. gingivalis* in the saliva indicates activation of the local immune response to this pathogen, which leads to inflammation in the periodontal tissues, causes epithelium ulceration in the periodontal pockets and may allow bacteria, their products and inflammatory mediators to reach the circulation, causing bacteremia (Hajishengallis 2015; Hajishengallis and Chavakis 2021). The presence of inflammatory mediators in periodontitis, such as IL-1, IL-1 β, IL-4, IL-6, IL-8, IL-10, TNF and IFN-γ, together with macrophages sensitized by *M. leprae* in the bloodstream can exacerbate the host’s immune response, triggering the leprosy reaction (Motta et al. 2011; Cortela et al. 2015).

Another finding of the present study is that in most samples, regardless of group, the salivary pH tended to alkalinity, which may represent a greater amount of bicarbonate in the saliva. In the presence of dental plaque biofilm and low salivary flow, this predisposes to the formation of dental calculus by the precipitation of calcium-bound phosphate from the biofilm, and consequently the development of inflammation (Andrade et al. 2015). Although the literature does not address the relationship between pH and salivary flow with the leprosy reaction, this study sought to identify if the behavior of these variables in individuals with leprosy could be related to a greater chance of developing a leprosy reaction among individuals with periodontitis, however, there was homogeneity between the case and control groups.

One of the strengths of the present study was the execution of logistic regression, in which age, level of education, and alcoholic beverage consumption were selected as confounders to neutralize the effect of these covariables. It is known that the age is an important covariable in the leprosy reaction with the disease being more frequent with advancing age.

In relation to the level of education, the lower number of years studied may reflect a lower practice of self-care and consequent decrease in the frequency of tooth brushing (Paulander et al. 2003; Márquez-Arrico et al. 2019). It should be noted however, that the physical disability related to the sequelae of the reactions can interfere with the development of motor activities (Barbosa et al. 2008; Monteiro et al. 2014), such as the practice of oral hygiene.

The finding that the consumption of alcoholic beverage was observed more frequently among individuals without a leprosy reaction, may represent information/memory bias since individuals are advised not to consume alcoholic beverage from the confirmation of the diagnosis of a leprosy reaction.

Some limitations of the present study can be pointed out: (1) specific antibodies for only one bacterium - *P. gingivalis*, were determined. Although *P. gingivalis* is considered a keystone pathogen of periodontal dysbiosis, it is possible that other microorganisms also involved in the pathogenesis of periodontitis may also reflect differences between the groups; (2) residual confounders, such as genetic factors and cell mediated immunity were not investigated; (3) total anti - *P. gingivalis* IgA antibody was measured and it is unknown how much of this was actual secretory - IgA. In addition, the present study did not aim to investigate the association between the periodontal disease, clinically diagnosed, and the leprosy reaction. Another investigation is being carried out to assess this possible relationship with a calculation of the minimum sample size suitable for evaluating this proposition.

Nevertheless, since *P. gingivalis* is a keystone pathogen in the periodontal dysbiosis (Hajishengallis 2015) and high titers of specific antibodies to this bacterium have been demonstrated in the serum and gingival crevicular fluid of subjects with periodontitis (Baranowska et al. 1989; Nakagawa et al. 1994), the findings of the present study may contribute to the understanding of the relationship between periodontal infection and the leprosy reaction using possible salivary markers. Further studies are required to identify other possible salivary markers as well as any association between the leprosy reaction and oral disease. The need for dental follow-up of individuals diagnosed with leprosy also needs to be explored as part of the monitoring of leprosy reactions.

## Data Availability

The data of this work are available at: http://www.labimuno.ufba.br/.
